# Severe Abdominal Pain in a Post-menopausal Obese Female

**DOI:** 10.4103/1319-3767.74447

**Published:** 2011

**Authors:** Vipul D. Yagnik

**Affiliations:** Ronak Endo-Laparoscopy and General Surgical Hospital, Patan -384265, Gujarat, India

A 56-year-old female came to emergency department with complaints of severe abdominal pain in the epigastric region with abdominal distention. She had also complained of fever. No significant past medical or surgical history was available. Her pulse rate and respiratory rate were 110/min and 26/min, respectively. On examination of abdomen, belly was distended with guarding and rigidity and periumbilical ecchymosis Figure 1. She was post-menopausal. Per-vaginal and per-rectal examination were normal.

**Figure 1:**
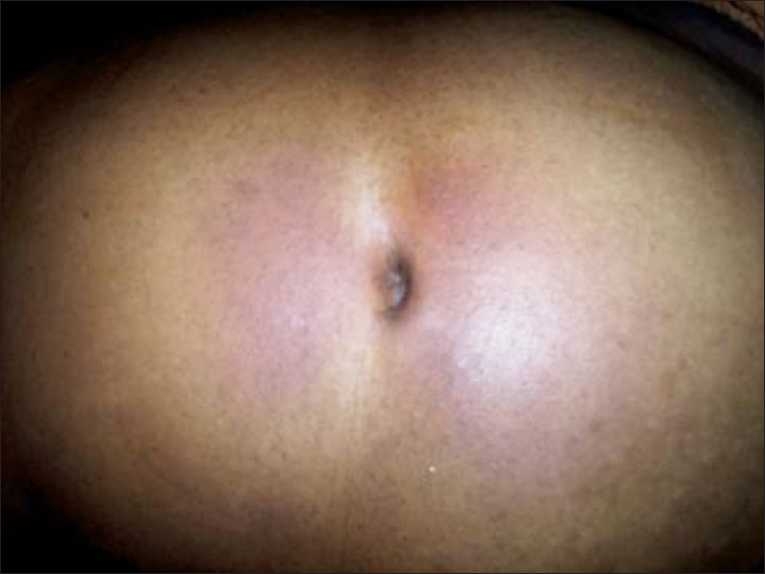
Periumbilical ecchymosis

## QUESTIONS

Q1. What is the sign known as and what is the differential diagnosis?
Q2. What is the mechanism of formation in acute pancreatitis?

## ANSWERS

**Table:** 

A1	Periumbilical ecchymosis is known as Cullen sign. Differential diagnosis for this sign includes: Acute hemorrhagic pancreatitis[1] Ruptured ectopic pregnancy[2] Blunt abdominal trauma Coagulation disorders Retroperitoneal hemorrhage Rectus sheath hematoma
A2	Methhemalbumin formed from the digested blood in a severe inflammatory process is responsible for Cullen sign.
